# Structured data sets of compounds with multi-target and corresponding single-target activity from biological assays

**DOI:** 10.2144/fsoa-2020-0209

**Published:** 2021-03-11

**Authors:** Christian Feldmann, Dimitar Yonchev, Jürgen Bajorath

**Affiliations:** 1 Department of Life Science Informatics, B-IT, LIMES Program Unit Chemical Biology & Medicinal Chemistry, Rheinische Friedrich-Wilhelms-Universität, Friedrich-Hirzebruch-Allee 6, Bonn D-53113, Germany

**Keywords:** biological assays, large-scale data analysis, machine learning, promiscuity, screening compounds, structural relationships

## Abstract

**Aim:**

Providing compound data sets for promiscuity analysis with single-target (ST) and multi-target (MT) activity, taking confirmed inactivity against targets into account.

**Methodology:**

Compounds and target annotations are extracted from screening assays. For a given combination of targets, MT and ST compounds are identified, ensuring test data completeness.

**Exemplary results & data:**

A total of 1242 MT compounds active against five or more targets and 6629 corresponding ST compounds are characterized, organized and made freely available.

**Limitations & next steps:**

Screening campaigns typically cover a smaller target space than compounds from the medicinal chemistry literature and their activity annotations might be of lesser quality. Reported compound groups will be subjected to target set-based promiscuity analysis and predictions.

## Lay abstract

The ability of a compound to bind to multiple biological targets by defined mechanisms is termed promiscuity. Analyzing compound promiscuity helps to better understand how drugs function that are capable of interacting with multiple therapeutic targets. In drug discovery, this phenomenon is referred to as polypharmacology. Machine learning using data sets of compounds with multi-target and corresponding single-target activity aids in identifying structural features that distinguish these compounds.

## Graphical abstract

Shown is an exemplary group of three compounds (CPDs) with single-target or multi-target activity. For grouping of compounds according to a set of targets, confirmed activity or inactivity against all targets is required.

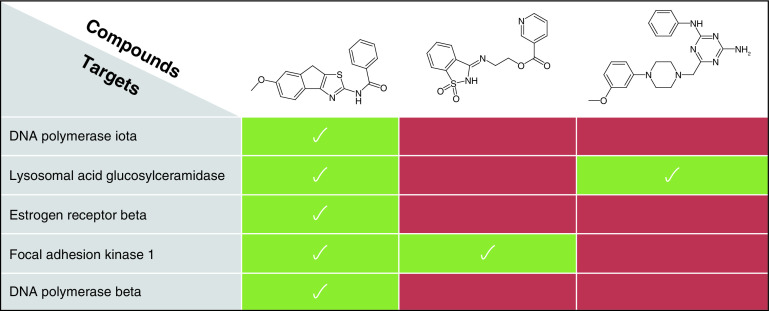

Compounds with multi-target activity (MT-CPDs) are also termed promiscuous compounds (without implying undesired properties) and play an increasingly important role in drug discovery for the treatment of complex and multifactorial diseases [1–3]. Promiscuous compounds are also of interest from a basic scientific perspective because they are capable of ‘pseudo-specific’ binding to multiple targets by engaging in ‘selectively nonselective’ interactions [4], the molecular basis of which is just beginning to be understood. Such ‘selectively nonselective’ interactions might be formed, for example, by inhibitors that are active against multiple kinases [4]. However, promiscuous compounds may also be active against distantly related or unrelated targets.

The design of MT-CPDs with predefined activities has become a topical issue in pharmaceutical research [5–7]. So far, MT-CPD design is mainly driven by combining pharmacophore elements of known single-target compounds (ST-CPDs) [5,6], also taking target (binding site) knowledge into account [5,6], and to a lesser extent by machine learning (ML) [7].

However, ML has been used successfully to classify promiscuous and nonpromiscuous compounds on the basis of chemical structure with reasonable to high accuracy [8–10]. These findings have been of particular interest because structural features that might generally distinguish between ST- and MT-CPDs are currently unknown. ML analysis has also revealed that nearest neighbor (NN) relationships between MT-CPDs on the one hand, and corresponding ST-CPDs on the other strongly contributed to successful predictions using different algorithms, with NN classifiers often approaching the accuracy of ML models [8–10]. Hence, the picture is emerging that MT-CPDs are often more similar to each other than to corresponding ST-CPDs and *vice versa*, hence providing a rationale for successful predictions and underlying structural relationships [9,10].

Meaningful promiscuity analysis and predictions require data sets with well-defined composition of MT- and ST-CPDs, the generation of which is laborious and requires careful curation of structures and activity data. Such data sets are useful for multiple purposes including *in silico* analysis of molecular promiscuity; prediction of MT-CPDs; exploration of structure-promiscuity relationships in medicinal chemistry; or selection of template structures for MT ligand design. Therefore, we make the data sets derived for our recent complementary compound promiscuity analyses [9,10] freely available in organized form to the computational and medicinal chemistry community. In this data note, we report these open access depositions and provide a detailed description of the dataset derived from screening compounds [10], hence enabling further use. For additional methodological details and background information, the interested reader is referred to the original publications [9,10].

## Methodology

### Assay selection criteria

Compounds and their activity (target annotations) were extracted from screening assays available in the PubChem BioAssay database [11]. Assays for individual targets specified by PubChem Gene Identifiers were selected, excluding assays sourced from other databases (such as ChEMBL, PDBind or Tox21). PubChem-external assays were excluded to avoid overlap in MT- and corresponding ST-CPDs in subsequent studies exclusively focusing on compounds from medicinal chemistry [9] and screening sources [10] (there is compound cross-fertilization between these databases). For the selected PubChem assays, target Gene Identifiers numbers were mapped to UniProt IDs [12]. At this stage, potentially ambiguous assays were omitted for which no target UniProt IDs were available as well as assays for nonhuman targets or known antitargets (such as hERG, CYP or P-glycoproteins). Compounds with activity against antitargets are not pharmaceutically relevant. Furthermore, assays with unusually high hit rates (>2%), any reported inconsistencies, or designated cytotoxic compounds were removed.

Compound promiscuity analysis is particularly vulnerable to false positive assay readouts and activity/target annotations. Therefore, filters for potentially liable pan assay interference compounds [13,14] as well as filters comprising empirical chemical liability rules [15] were applied to flag and remove potential interference compounds, which often give rise to artifacts across different assay formats. Furthermore, likely colloidal aggregators [16] and confirmed firefly luciferase (FLuc) inhibitors [17], which might also give rise to assay artifacts, were removed. For the analysis of MT-CPDs, no potency threshold was applied.

**Figure 1.**
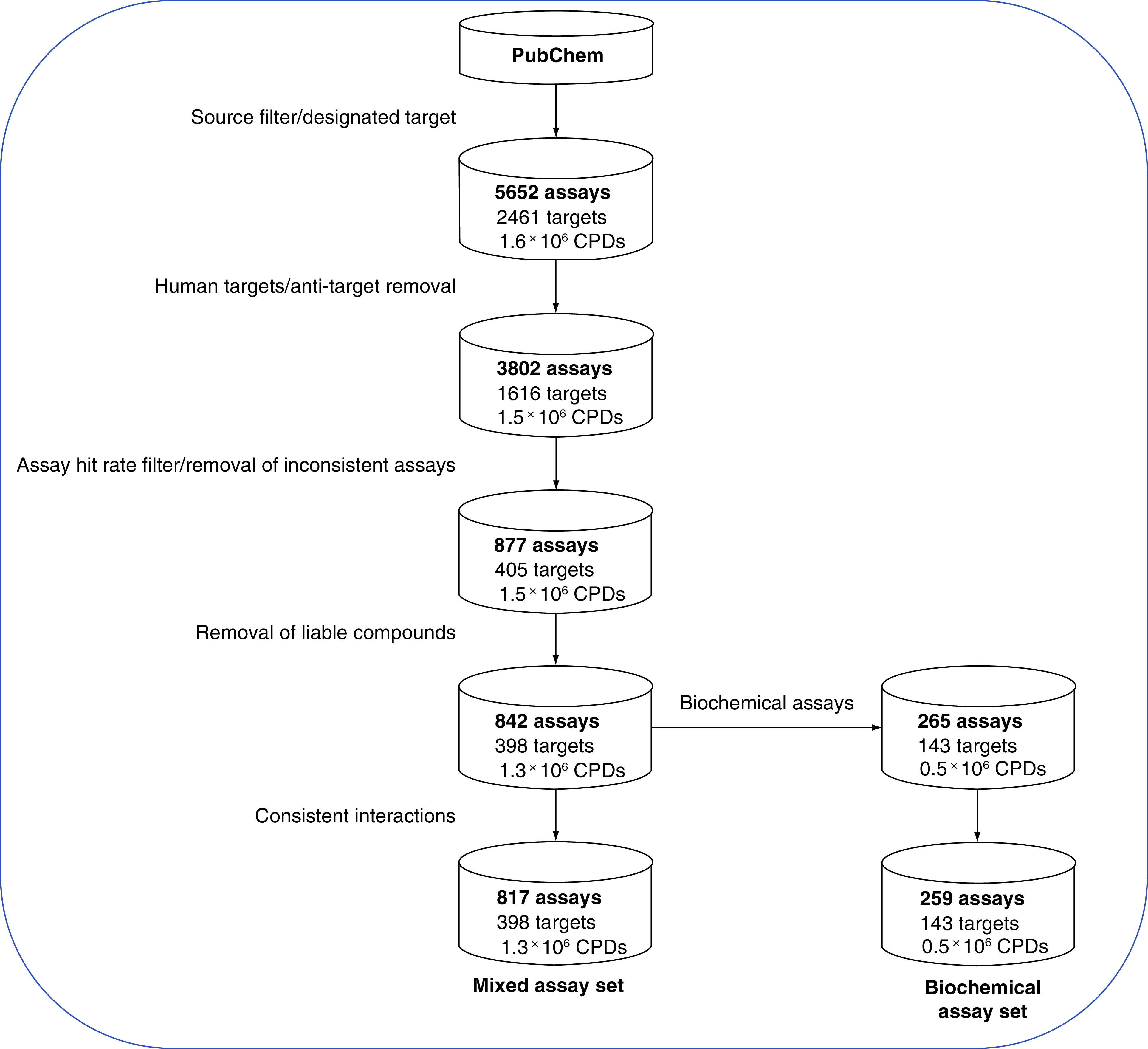
Assay data curation. The workflow applied to select assays and compounds from PubChem for our data sets is summarized. Two assay sets were assembled. Biochemical assays represented a subset of mixed assays. CPDs: Compounds.

The final assay selection contained both biochemical and cell-based ST assays and was termed the “mixed” dataset. From this set, the subset of biochemical assays was selected and separately analyzed. From both the mixed and biochemical dataset, small numbers of assays containing inconsistent compound-target annotations were removed, yielding our final selection sets. The data curation workflow and associated statistics are summarized in Figure 1. The final mixed and biochemical assay set covered 817 and 259 different assays and 398 and 143 diverse biological (protein) targets, respectively.

### Compound grouping & sampling strategy

The ‘promiscuity degree’ (PD) of each compound from the mixed and biochemical sets was calculated as the number of targets the compound was active against. Importantly, for the biochemical assay set, compound PD values were generally lower than for the mixed set, due to the smaller number targets associated with the biochemical set. For promiscuity predictions, the choice of varying PD levels provided a control [10]. PD ≥5 was applied as a criterion for compounds with significant promiscuity and PD ≥3 as a control. For PD ≥5, 1248 and 2886 MT-CPDs were obtained for biochemical and mixed assays, respectively. For PD ≥3, an additional set of 3590 MT-CPDs was obtained for biochemical assays.

 The final data sets were assembled by pairing MT-CPDs with one or more ST-CPDs (PD = 1) from the same assay set. For each target of a given MT-CPD, an ST-CPD that was active against the target and experimentally confirmed to be inactive against the remaining targets was selected. According to this selection strategy, an ST-CPD was selected only once. Depending on the number of targets per MT-CPD, a qualifying ST-CPD might not be available for each target, which did not preclude the formation of a compound group if ST-CPDs were available for other targets involved. MT-CPDs without corresponding ST-CPDs were omitted. This selection scheme ensured that experimental data for each group of MT- and corresponding ST-CPDs were complete; in other words, each compound was tested against all targets of a group. Therefore, PD values were not potentially underestimated due to missing test results, which might occasionally cause false negative MT-CPDs (erroneously classified as ST-CPDs). The availability of experimental test frequencies of compounds as well as positive and negative assay results was a prime motivation for generating sets of MT- and ST-CPDs on the basis of assay data. The composition of the obtained datasets is reported in Table 1. Test frequencies and negative assay results are not available for compounds from medicinal chemistry sources (such as those collected in ChEMBL).

**Table 1.** The composition of compound datasets from mixed and biochemical assays reported for different promiscuity degree thresholds.

Dataset	Mixed	Biochemical	
	PD ≥5	PD ≥5	PD ≥3
MT-CPDs	2858	1242	3468
ST-CPDs	15,839	6629	11,793

MT-CPD: Compound with multi-target activity; PD: Promiscuity degree; ST-CPD: Single-target compound.

### Reduced data sets

To assess the influence of NN relationships on the predictions, two equally sized compound subsets were sampled from each dataset. The first subset was obtained by randomly removing 50% of the original compound groups (random removal), whereas for the second subset, 50% of most similar compound groups were systematically eliminated (NN removal). First, pairs of groups were formed and sorted by most similar ST-CPDs. From the two groups of the top ranked pair (highest calculated fingerprint similarity), the group with higher similarity to the next most similar group was removed. The process was repeated until 25% of the compound groups were eliminated. Additionally, this procedure was applied to MT-CPDs, resulting in a final dataset containing only 50% of the original compound groups. Accordingly, this reduced dataset was obtained by iteratively removing NN groups, which served as another control for promiscuity predictions.

## Exemplary results

### Test frequencies

**Figure 2.**
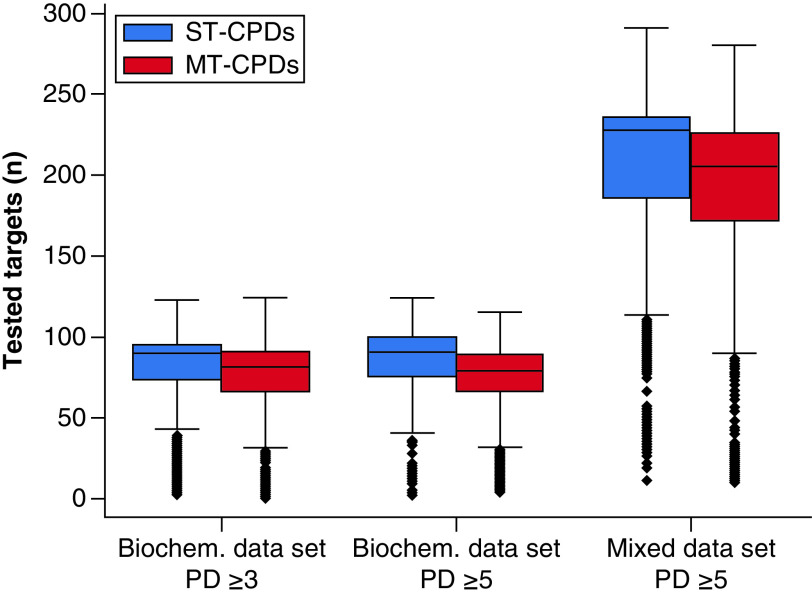
Compound test frequencies. Boxplots report test frequency distributions of ST- and MT-CPDs in the respective datasets. Each distribution is represented by its minimum (lower whisker), lower quartile (lower boundary of the box), median (horizontal line in box), upper quartile (upper boundary of the box) and maximum (upper whisker). Values classified as statistical outliers are shown as diamonds. MT-CPD: Compound with multi-target activity; ST-CPD: Single-target compound.

The probability of a compound to exhibit MT activity statistically increases with the number of targets it has been tested against. Therefore, controlling test frequencies for ST- and MT-CPDs is an important step for promiscuity analysis. Figure 2 reveals that ST-CPDs had higher test frequencies compared with MT-CPDs in all three data sets, thus lending further support to the MT- versus ST-CPD assignments.

### Compound sampling

**Figure 3.**
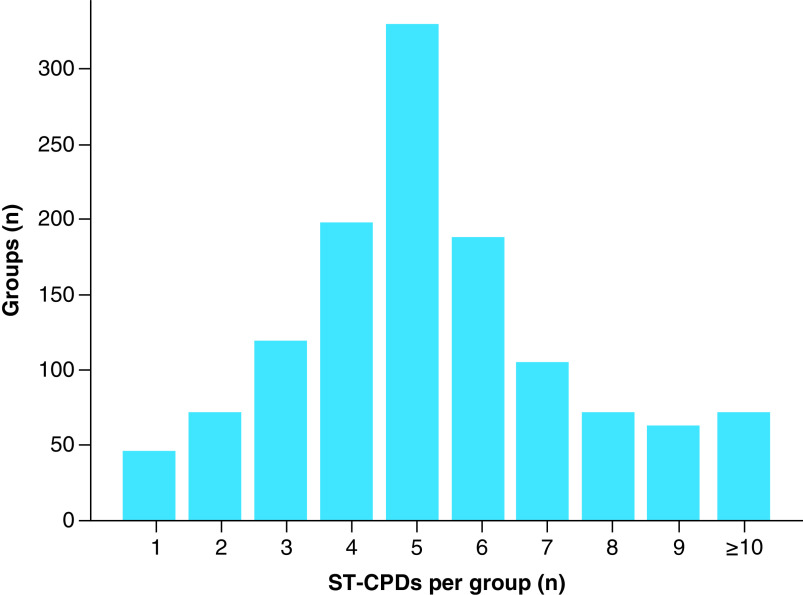
Single-target compounds per compound with multi-target activity. Histogram bars report the number of ST-CPDs sampled for each MT-CPD (biochemical set, MT-CPDs: PD ≥5). MT-CPD: Compound with multi-target activity; ST-CPD: Single-target compound.

The quality of the datasets also depended on the availability of corresponding experimentally confirmed ST-CPDs for given MT-CPDs. Figure 3 shows a representative distribution of ST-CPDs per MT-CPD. For the majority of MT-CPDs at least five corresponding ST-CPDs were available, yielding reasonably sized compound groups.

### Predictions

**Figure 4.**
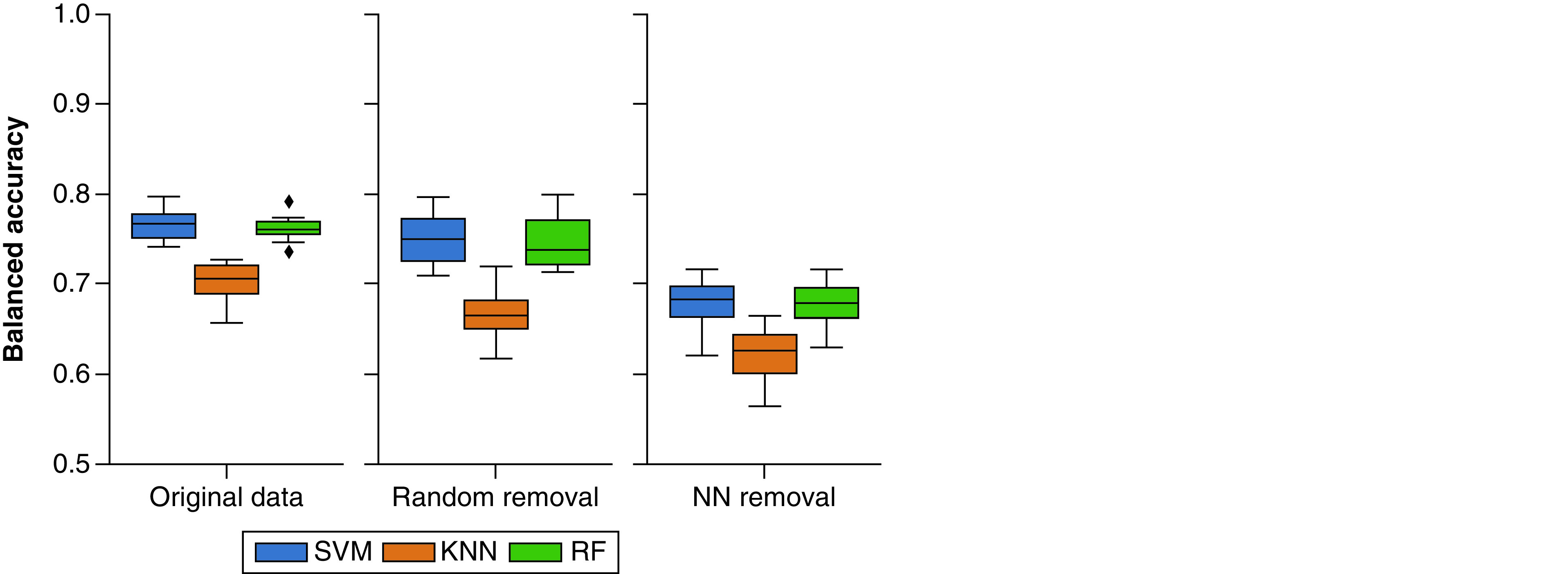
Prediction accuracy. Boxplots report distributions of balanced accuracy values for SVM (blue), k-NN classifier (KNN, orange) and RF (green) predictions of MT- versus ST-CPDs over 10 individual trials for the original dataset (biochemical set, MT-CPDs: PD ≥5, left) and reduced data sets after random compound removal (middle) and NN removal (right). MT-CPD: Compound with multi-target activity; NN: Nearest neighbor; RF: Random forest; ST-CPD: Single-target compound; SVM: Support vector machine.

Representative results for predictions distinguishing between MT- and ST-CPDs are shown in Figure 4. Prediction accuracy using different ML methods and a k-NN classifier was generally high for original data sets. Random removal of 50% of compound groups only led to a small reduction in predictive performance, whereas NN removal resulted in a larger reduction in prediction accuracy, revealing the importance of NN relationships between MT- and ST-CPDs, respectively [10].

## Data

### Composition

The data curation and compound selection protocols described above yielded three datasets of well-defined composition for promiscuity analysis. The mixed dataset comprised compounds from both biochemical and cell-based assays with an MT-CPD threshold of PD ≥5. From the subset of biochemical assays (having higher ST confidence than cell-based assays), two datasets were generated with MT-CPD thresholds of PD ≥5 and PD ≥3, respectively. Application of the lower PD threshold further increased dataset size. From each of these two biochemical sets, two reduced datasets containing half of the compound groups were generated through random compound or NN removal, respectively.

While the original datasets are suitable for multi-purpose promiscuity analysis, as specified above, reduced datasets were mainly designed for our ML studies [10]. The three original datasets are made available in an organized form, as further specified below. These datasets complement conceptually similar MT- and ST-CPD collections from the medicinal chemistry literature, for which no test frequencies and negative assay data (inactive compounds) were available. The earlier derived data sets have been described in detail [9] and made available on the Zenodo open access platform [18]. By design, these two specifically derived and well-structured open access data collections, which have yielded equivalent results in large-scale promiscuity analysis and predictions, complement each other.

### Data deposition

Three screening compound datasets are provided as tab-delimited text files (.tsv format). Each file contains canonical SMILES representations of the compounds (“NostereoAromaticSMILES”), their class label (”is_MT”), IDs for grouped compounds (“group”), PubChem compound IDs (“cid”), concatenated lists of corresponding target IDs (“target_ids”) and the number of experimentally tested targets (“n_tested”). In addition, randomly removed compounds (“random_removal_set”) and compounds removed based on NN relationships (“nn_removal_set”) are identified such that the reduced data sets can be immediately extracted. As a part of this study, the datasets are made freely available as a new deposition on the Zenodo open access platform [19].

## Limitations & next steps

The only limitations of the current datasets are the intrinsic variability of screening assay data and lower-confidence activity measurements compared with compounds for which equilibrium constants have been determined. However, for promiscuity analysis and the exploration of structure-promiscuity relationships, qualitative target annotations are sufficient and exact potency values are not required. We will further use the datasets reported herein for analyzing differences between MT- and corresponding ST-CPDs for different target combinations, representing a form of ‘local’ promiscuity analysis, setting it apart from global assessment and predictions across all targets. We hope that these datasets will also be useful to others for exploring different facets of compound promiscuity from a computational or medicinal chemistry perspective.

**Executive summary**

**Introduction**

- Compounds with multi-target activity are introduced.
- Compound promiscuity is discussed in the context of polypharmaology.

**Methodology**

- Data curation protocols are detailed.
- Compound selection and sampling strategies are explained.
- The value of test frequencies and negative assay results is emphasized.
- Dataset design is rationalized.

**Exemplary results**

- Test frequencies are analyzed.
- Ratios of multi-target versus corresponding single-target compounds are quantified.
- Exemplary promiscuity predictions are reported.

**Data**

- Different datasets are described.
- The data deposition is detailed.

**Limitations & next steps**

- Assay data variance is discussed.
- Further refined promiscuity analysis is proposed.
